# Produced Water Treatment with Conventional Adsorbents and MOF as an Alternative: A Review

**DOI:** 10.3390/ma14247607

**Published:** 2021-12-10

**Authors:** Humaira Gul Zaman, Lavania Baloo, Rajashekhar Pendyala, Pradeep Kumar Singa, Suhaib Umer Ilyas, Shamsul Rahman Mohamed Kutty

**Affiliations:** 1 Civil and Environmental Engineering Department, Universiti Teknologi PETRONAS, Seri Iskandar 32610, Malaysia; humaira_19001707@utp.edu.my (H.G.Z.); shamsulrahman@utp.edu.my (S.R.M.K.); 2 Chemical Engineering Department, Universiti Teknologi PETRONAS, Seri Iskandar 32610, Malaysia; raj.pendyala@gmail.com; 3 Department of Civil Engineering, Guru Nanak Dev Engineering College Bidar, Bidar 585403, India; pmsinga@gmail.com; 4 Institute of Hydrocarbon Recovery, Universiti Teknologi PETRONAS, Seri Iskandar 32610, Malaysia; suhaibui@outlook.com

**Keywords:** adsorbents, adsorption, metal–organic frameworks (MOFs), produced water, sustainability, water treatment

## Abstract

A large volume of produced water (PW) has been produced as a result of extensive industrialization and rising energy demands. PW comprises organic and inorganic pollutants, such as oil, heavy metals, aliphatic hydrocarbons, and radioactive materials. The increase in PW volume globally may result in irreversible environmental damage due to the pollutants’ complex nature. Several conventional treatment methods, including physical, chemical, and biological methods, are available for produced water treatment that can reduce the environmental damages. Studies have shown that adsorption is a useful technique for PW treatment and may be more effective than conventional techniques. However, the application of adsorption when treating PW is not well recorded. In the current review, the removal efficiencies of adsorbents in PW treatment are critically analyzed. An overview is provided on the merits and demerits of the adsorption techniques, focusing on overall water composition, regulatory discharge limits, and the hazardous effects of the pollutants. Moreover, this review highlights a potential alternative to conventional technologies, namely, porous adsorbent materials known as metal–organic frameworks (MOFs), demonstrating their significance and efficiency in removing contaminants. This study suggests ways to overcome the existing limitations of conventional adsorbents, which include low surface area and issues with reuse and regeneration. Moreover, it is concluded that there is a need to develop highly porous, efficient, eco-friendly, cost-effective, mechanically stable, and sustainable MOF hybrids for produced water treatment.

## 1. Introduction

Oil and gas reserves play an essential role in the global economy, and resource use has been intensified over the years to meet rising energy demands [1]. Oil and gas industries produce a significant amount of wastewater in large quantities, bringing it to the surface as part of the oil production process. This wastewater is generally termed produced water (PW). It is polluted with heavily immiscible oil, organics, heavy metals, salts, suspended solids, and radioactive components [2]. From 1990 to 2015, PW production increased from less than 30,000,000 barrels per day to approximately 100,000,000 barrels per day [3]. Worldwide, day-to-day fuel consumption is expected to increase from 85 million barrels in 2006 to 106.6 million barrels by 2030 [4]. The oil and gas production activities have produced a vast amount of PW, with oil generating a greater upward flow of PW compared to gas wells [5]. It has been estimated that offshore oil and gas fields generate 39.5 Mm^3^/day of PW [6]. Globally, the volume of PW production is approximately 200 Mbbl/day, which is three times more than oil production [7].

Furthermore, the PW volume in depleted fields could reach as high as 98% of the remaining content, with only 2% of what is recovered being suitable for fossil fuel production [8]. Untreated PW discharge into the ecosystem may have a disastrous effect on the environment due to the high levels of hazardous contaminants. Therefore, the oil and gas industries must treat PW to protect the environment and human wellbeing. 

Several methods of treating PW have been investigated in the literature, some of which involve using physicochemical, biological, and membrane technologies [2,9,10,11,12]. Conventional treatment technologies, such as physical methods, require a long retention time, a vast land area, high initial costs, and secondary pollutant production. On the other hand, chemical methods require high chemical inputs and uneconomical operational procedures. Moreover, the secondary pollutants, in the form of sludge, need further treatment, increasing the treatment costs. Physicochemical treatments only transfer the contaminants from one medium to another without complete degradation and mineralization. The literature suggests that conventional treatment methods have low remediation efficiencies as standalone technologies [13]. For instance, membrane technology can only perform better when integrated with biological processes. It also has several disadvantages, such as high energy pressure requirements, membrane fouling, and high maintenance costs. The drawbacks limit these methods’ applicability at the commercial level because industries require efficient, economical, and environmentally friendly treatment processes. The traditional techniques reinject waste streams into the well, discharge it directly, or reuse waste in a thermal loop. Thus, there is a need for alternative methods and technologies for PW treatment. Adsorption is one of the most attractive environmental remediation techniques due to its design simplicity, its low operational cost, and its minimization of chemical or biological sludges. It can be applied without high temperature or pressure requirements and may remove harmful chemicals and pollutants from the environment [14]. In adsorption mechanisms, a solid surface comes into contact with liquids and tends to accumulate a surface layer of solute molecules, which provides complex and compelling pollutant-reduction abilities. It is reported that adsorption could easily remove 80% of the heavy metals from PW and be capable of restoring 100% of water [10].

However, adsorption is still being utilized merely as a unit process or polishing step in the treatment train. Yet strict water quality parameters can be met efficiently through adsorption. The most important advantages of using the adsorption process are its operational ease, the regeneration of the adsorbent, and the fact that is has no additional chemical requirements. Various adsorbent materials have been studied for use in adsorption methods, such as nanomaterials, nanocomposites, nanoparticles, clays, biopolymers, metal–organic frameworks (MOFs), and zeolites [15,16,17,18]. In the last decade, MOFs, as a novel type of exceptionally crystalline porous materials that are assembled by connecting ligands with metal ions or metal ion clusters, have become the focus of a significant amount of research [17,19]. MOFs have remarkably large surface areas, porous sizes, shapes, and structures. Furthermore, because the functional groups of these materials influence their adsorption characteristics, MOFs can be functionalized, making them promising adsorbents [17].

This review paper focuses on evaluating the feasibility of the adsorption process, given that it is one of the most promising techniques for removing pollutants. However, to the best of our knowledge, there have been no comprehensive reviews that have holistically evaluated the efficiency and suitability of different conventional adsorbent methods when used with PW, nor any which have discussed developments in the creation of highly porous, reusable adsorbents. Future opportunities to use modern, highly porous, reusable adsorbents, which may be an alternative to conventional ones, are highlighted. Currently, a limited amount of literature is available on the application of adsorption techniques for PW treatment. This review critically analyzes the suitability and limitations of different available adsorbents for PW treatment. Recent progress and developments in applying MOF adsorbents in PW treatment are emphasized. Future perspectives on PW treatment using eco-friendly, cost-effective, and reusable adsorbents are pointed out, which some believe can decrease our dependence on limited freshwater resources. 

## 2. Composition of Produced Water

Water accounts for the majority of the composition of PW. Its composition varies considerably based on the geographical location, reservoir characteristics, hydrocarbon production, and minerals in the water-bearing geological formation [20]. PW consists of organics, inorganics, production chemicals, and transformation compounds, which are outlined in Table 1 [21]. The organic compounds in PW are oil and grease, as well as aromatic compounds, including benzene, toluene, ethylbenzene, and xylenes (BTEX). BTEX have a non-polar aliphatic character and interact with other substances through weak dispersive forces [22]. PW may contain critical organic pollutants, such as halogenated aromatic compounds, chloroform, naphthalene, phenanthrene, dibenzothiophene (NPD), polyaromatic hydrocarbons (PAHs), phenols, and trichloroethylene.

PW salinity (salt concentration) can range from 1000 mg/L to more than 300,000 mg/L, whereas seawater salinity ranges from 32,000 mg/L to 36,000 mg/L. Chloride and sodium are the anion and cation with the greatest concentrations in PW, similar to those found in seawater. PW also contains soluble salt ions, such as calcium, magnesium, potassium, and bicarbonate. Sulfide is present in PW that originates from sulfur-bearing oil and gas sources and wells. Corrosion issues that stem from the high concentrations of salts, sulfurs, and sulfides should thus be considered, particularly for offshore PW treatment equipment. The inorganic composition of PW, including, significantly, heavy metals, is determined by the formation conditions and the chemicals injected into oil and gas reservoirs during exploration and production. Other elements, such as barium, iron, manganese, and zinc, are known elements that might be detected in PW which are not typically present in saltwater. These metal ions, which form undesired solids, may hinder the treatment processes [23,24]. The difference in quantities of these ions between PW and saltwater may be one of the primary causes of PW’s high potential biological toxicity. Moreover, some anaerobic bacteria also exist in PW and can cause corrosion. The PW from oil fields and natural gas vary in terms of composition and concentration [10,13,25].

### 2.1. Concentrations of Radioactive Compounds in Produced Water

In PW, both naturally and artificially occurring radionuclides are present because of anthropogenic activities. They are referred to as technologically enhanced, naturally occurring radioactive materials (TENORM). Their presence may be caused by uranium and thorium chains [23]. The water that is utilized in the production process may contain a specific amount of radioactive materials, such as 232 Th, 238 U, 226 Ra, 228 Ra, 210 Pb, 224 Ra [24]. Because of the presence of TENORMs, PW has radioactivity impacts on the environment and living organisms [26]. Leakages of TENORMs might have significant environmental impacts. Hence, it has received a lot of interest globally in recent years. The radioisotope values of PW in several regions of the world are presented in Table 2.

### 2.2. The Impact of Produced Water on the Environment

Environmental impacts caused by PW disposal since the mid-1800s have been reported after the first oil and gas wells were drilled and operated. The most commonly reported environmental concerns are soil degradation, and its effects on surface water, groundwater, and the ecosystem [50]. PW compounds are more hazardous and environmentally harmful than crude oil, and they may contaminate natural resources. Hazardous substances in high quantities are the most serious environmental issue when PW is discharged into the natural environment. Since produced water from offshore oil and gas production is mostly discharged into the ocean, it can impact the natural ecosystem and expose living organisms to harmful elements. PW is treated with gravity-based separation before being released into the environment. The discharge effects depend on a specific environment’s physical, chemical, and biological composition because PW contains high levels of dissolved ions, hydrocarbons, and trace elements. The untreated PW discharge poses a significant threat to aquatic life and agricultural resources by altering the aquatic environment’s natural state [13,51]. For agricultural purposes, high sodium and high conductivity in PW require further treatment to eliminate the risk of damaging crops and livestock. Large PW volumes cause environmental impacts, including the erosion of large land area disposal basins, pipelines, and road infrastructures. The size of the water body receiving the PW is critical in determining the environmental effects, as the ocean offers substantive dilution of discharge, while small streams have low dilution capacity.

The water body’s physical properties are of primary concern, including its temperature, pH, effervescence, and dissolved oxygen concentrations, which can be affected differently depending on the type of well that the PW originated from.

Non-polar oil in water (OIW) has been regulated by the government, whereas little attention has been given to the dissolved organic components in produced water [14,52]. Recent research has attempted to determine the long-term effects on the environment caused by the dissolved organic components, heavy metals, and production chemicals. Their effects are not fully documented or understood. Highly alkylated phenols, aromatic hydrocarbons, and a few metals are highly toxic to the environment, even at low concentrations, and cause bioaccumulation and toxicity [53]. The high concentration of salts in PW contributes to its toxic effects on soils, water quality, and ecosystems [54]. PW has higher salinity than seawater, and it destroys the quality of freshwater and degrades the quality of soil. High sodium levels can inhibit water filtration through the soil and may cause a deficiency of other essential ions required for growth [55,56].

A previous study [57] reported that the amount of fatty acid that organic matter contains generates a high level of biological oxygen demand (BOD) and chemical oxygen demand (COD). In PW, most metal ions are in a dissolved form and are chemically reactive, but the metal concentration is slightly higher than seawater [58]. This can have an adverse effect on the receiving water body and aquatic life due to its bioaccumulation potential, and it may therefore harm biological communities [58,59]. Boron, lithium, bromine, fluorine, and radium are present in elevated concentrations, and these trace elements may even remain in soils after the saline water has been flushed away. Many trace elements are phytotoxic and are adsorbed in the soil. Schifter et al. [60] studied the effect of produced water effluents in Sonda de Campeche, located in the Gulf of Mexico. The study was based on the direct field sampling of effluent that had been released into the ocean in the years 2003–2013. The results showed that the sum of the average metal concentration increased from 272–1104 µg/L over 2003–2013.

**Figure 1:**
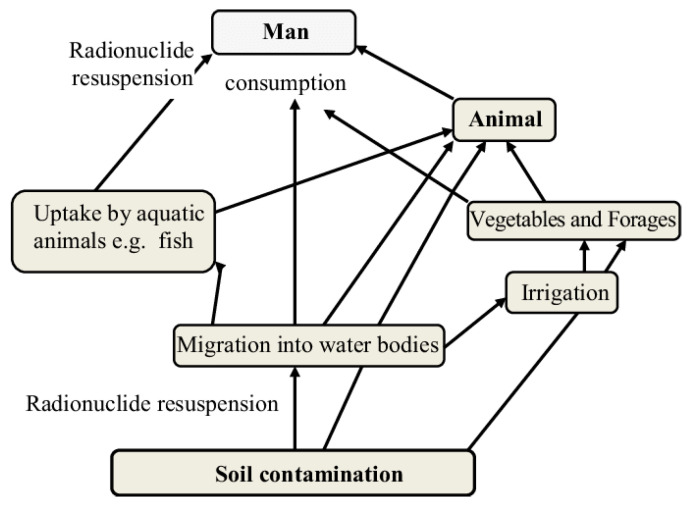
Radionuclide entry into the environment [67].

A broad range of natural and artificial radioactivity is present in PW. Thus, their harmful effects on the environment vary from region to region, depending on how radionuclides enter the environment. This variance is represented in Figure 1. The bioaccumulation of 226 Ra in the food series is a possible risk for living organisms; therefore, the ecosystem must be protected from this threat. The potassium isotope is a naturally occurring radioactive element, and it is being released in large quantities and mostly appears as a radionuclide in PW. Previous studies [37,61,62,63,64] reported that 34% of 40 k isotope activity concentrations were found to be less than 20 Bq.L^−1^ and approximately 25% ranged between 20 and 60 Bq.L^−1^. A high concentration of radioisotope in PW might lead to the contamination of agricultural soil and groundwater. The lead isotopes 214 Pb and 210 Pb are present in PW, and lead isotope activity concentrations are less than 20 Bq.L^−1^ [65]. Lead isotopes, after release, degrade the soil and groundwater quality. The actinium (228 Ac) isotope poses a considerable long-term health risk due to its well-known toxicity at high levels [66]. The cesium 137 Cs artificial radionuclide has also been detected in Iraqi oil PW because of the Gulf War and the Iraq War [44]. Previous studies [32,33,35,63] reported the presence of other radioisotopes at detectable levels, including 137 Ba, 210 Po, 212 Bi, 214 Bi, and 208 Ti, but with low activity concentrations in comparison to the aforementioned radioactive elements [63,66]. Recent research worldwide aims to highlight the need for ecosystem protection. Therefore, an appropriate preventative policy is required to address environmental issues, and protect against harmful organics, chemicals, and radioactive materials.

### 2.3. The Impact of Produced Water on Human Health

**Figure 2:**
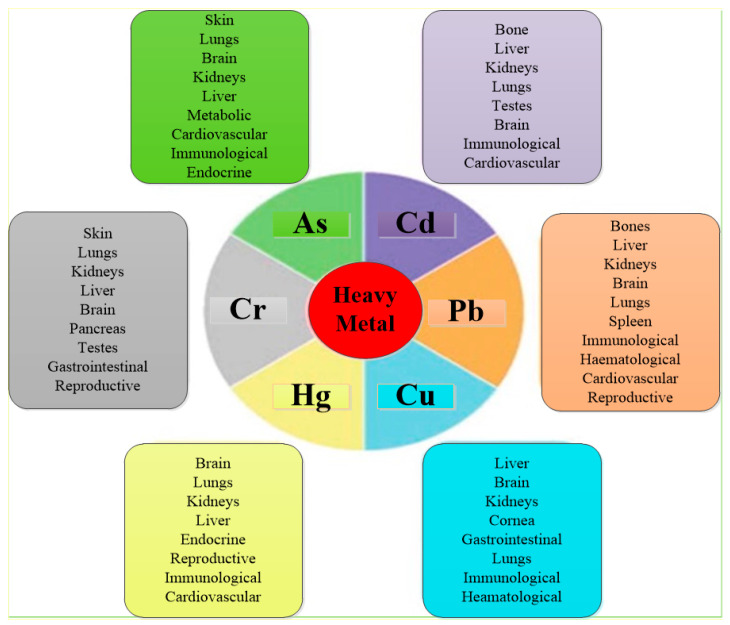
Harmful effects of heavy metals on human health. Figure was reproduced from [68].

The increase in produced water volumes across the world and its ongoing consequences impact the lives of present and future generations. The use of contaminated water has several negative impacts on human health. Significantly, it causes typhoid, cholera, hepatitis, and various other disorders. Despite the toxic composition of PW and its effects on human health, minimal focus has been given to the issue, and limited studies have been conducted. Contamination by heavy metals has proven to be a severe problem with several health hazards. According to the Agency for Toxic Substances and Disease Registry, metals and metalloids, such as As, Cr, Cd, Ni, Cu, Zn, and Pb, are ranked on the 275 extremely toxic pollutants list. Overall, the harmful effects of toxic heavy metals are summarized in Figure 2.

Previous studies [52,69,70] have reported that metals and hydrocarbons from oil fields are toxic, showing how exposure to alkylphenols has negative impacts on both the organs and fertility of fishes. Toxicity in produced water can be either acute or chronic. The LC 50 test is used to measure acute toxicity, whereas the long-term effects are more difficult to quantify [13,59]. Holdway et al. [71] argued that long-term chronic exposure could cause growth and developmental problems, fecundity, genetic diversity, and lower reproductive success. It may also cause respiratory issues, physiological disorders, and endocrine disruption. Despite this, radium-bearing scales and sludge found in oilfield equipment are being discarded on soils, which poses additional hazards to human health.

The presence of a wide range of radioactivity in PW makes it critical to consider the effects on public health. Radionuclide emissions and their long-term persistence in the environment might cause long-term external irradiation or internal contamination via food or water consumption for populations living in affected regions. Radionuclides and their compounds are harmful in two ways. Chemical toxicity is induced because of the chemical characteristics of the elements and molecules that make up the material. Radiotoxicity refers to the toxicity of radioactive elements and nuclides. Inhalation and ingestion of a small number of radium isotopes (226 Ra, 228 Ra, and 224 Ra) may lead to their accumulation which can cause serious harm over the long term. Continuous exposure to radium results in bone and sinus cancer.

Moreover, they can potentially cause common diseases, such as nasal mucosa and bone tumors [72]. One investigation [49] reported that PW’s uranium and thorium isotope activity concentrations are less than 20 Bq.L^−1^. Water polluted with depleted uranium or thorium will undoubtedly cause many diseases to the consumer due to its penetration into the soil and water [73].

### 2.4. Produced Water Management—Discharge

Nowadays, managing PW requires considering all the factors while making decisions regarding management alternatives. Oil and gas companies face significant technical and economic challenges in the disposal and management of PW. The fundamental differences between offshore and onshore PW management are weight and space constraints, which impacts total treatment efficiency. Different management systems are also influenced by differences in environmental regulations and standards, as well as differences in the production volume and targeted pollutants. While onshore operations focus on reducing salt content, oil and grease levels are the primary concern in offshore operations [3]. Stream management is necessary to manage the production of hydrocarbon waste. The cost of managing PW varies according to the operational techniques. A technique for PW management that is both economical and eco-friendly would be considered to be the best practice in all industries.

**Figure 3:**
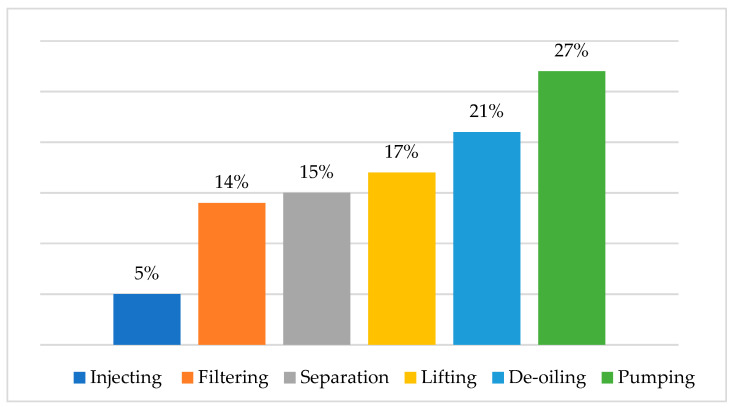
The produced water treatment and disposal life cycle cost in the USA (data obtained from [78]).

The United States produces approximately 890 billion gallons of PW annually [74] from seven key oil and gas basins [75]. With the unconventional nature of oil and gas development, the PW volume generated each year keeps increasing, with levels reaching more than 50% of the amount of crude oil and natural gas [76]. Reinjection of more than 55% of the PW into wells is common nowadays, but this means that the PW rests on open surfaces [77,78]. The global estimated treatment cost of PW is USD 40 billion per annum, and the disposal cost depends on the method, but typically ranges between 0.3–10 USD/bbl [79]. PW treatment with conventional techniques, such as hydro cyclone, media filters, and gravity separation, could generate treated water for reinjection at the cost of 0.509 USD/m^3^ of water. For PW recycling, the improved technique will produce recyclable water at the cost of 3.808 USD/m^3^ of water [80]. Figure 3 shows that the average costs percentage in the USA for handling water production [78].

To regulate effluent discharge, countries have different environmental and federal regulations and limits for the content in PW [81], as presented in Table 3. For offshore PW, only oil and grease content is regulated because the ocean has a high salt concentration, so salinity is not a concern. For onshore PW, both oil and salt parameters are regulated. For reinjection, then, oil content, solids, and bacteria must be eliminated. The limits on the amount of oil permitted in water vary from country to country. Different nations have set strict environmental rules and requirements for the discharge of produced water. The daily maximum limit for oil and grease is 42 mg/L, and the average monthly limit is 29 mg/L, according to the EPA’s effluent regulations for oil and gas extraction [82] (Table 3). After complying with environmental rules and standards using treatment equipment, PW from offshore oil and gas operations is often discharged directly into the ocean. At the same time, onshore operations manage the vast majority of PW by injecting it back into the wells. What little else remains is released, reused, or evaporated [83].

### 2.5. The Reuse of Produced Water

Another potential alternative in water management is the reuse of produced water, aiming to minimize demand for water. Produced water is a reusable resource that can be utilized for various purposes, i.e., drinking water, irrigation, industrial uses, and livestock watering. The EPA has provided standards that are more stringent for drinking water and, therefore, more extensive PW treatment is needed [86]. Additionally, to reuse treated water for irrigation and livestock, standards have been laid out by the US Department of Agriculture Natural Resources Conservation Service [87]. It may be used in the industry for various purposes, including platform cleaning and ship balancing. PW might therefore meet the water requirements of many industrial operations in water-stressed areas.

## 3. Produced Water Treatment

Several technologies for PW treatment have been proposed that focus on different contaminants. Previously, various methods have been reported for produced water treatment [7,88,89,90], such as physicochemical and biological methods. A series of individual unit processes are required for contaminant removal instead of a signal process, which might not remove all pollutants. Treated PW could be reused for industrial and agricultural purposes. PW contains varying concentrations of different contaminants (oil and grease, dissolved gases, radioactive materials, metals, organics, solids, salts, and microorganisms), and it is challenging to select a suitable treatment method [91]. Treatment costs largely depend on the quality of the influent, energy costs, and plant capacity. The quality of the effluent and the cheapness of the method must be taken into account when considering appropriate PW treatment [92]. Due to the space constraint and equipment weight capacity, offshore PW treatment is challenging. PW treatment requires different treatment steps to remove contaminants. PW volume increases day by day, and other techniques have been reported in the literature for its treatment, such as membrane filtration, adsorption, and chemical precipitation. Determinations on the most cost-effective treatment and the desired water quality standards for reuse or discharge can change the selection of appropriate techniques. The freshwater shortage is increasing globally; PW could therefore be a vital water source after appropriate PW treatment. In the next subsections, adsorption is discussed in detail.

### 3.1. Adsorption Classification

**Figure 4:**
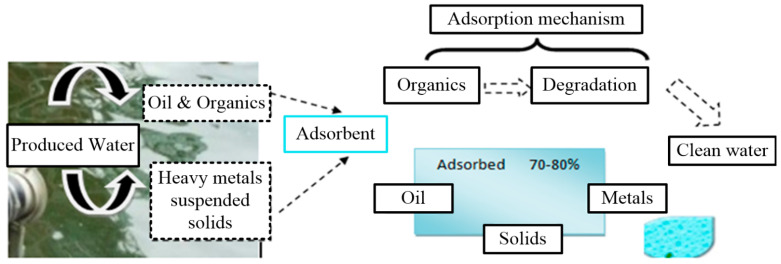
Adsorption mechanism for produced water contaminants.

Adsorption is considered to be an old, cheap, and much-improved technique that can help improve quality water [93]. Adsorption is an exothermic process, and its mechanism involves the attachment of either gas or solid substances onto an adsorbent surface, as shown in Figure 4.

Adsorption is typically divided into two categories: physisorption and chemisorption. This categorization is determined by the strength of the interaction between the substrate and the adsorbate. During isotherm and kinetic studies, this interaction has been identified. For example, if the kinetic model is fitted to a pseudo-second-order model, it posits that one adsorbate ion can occupy two surface sites; this suggests that the adsorption is classified as chemisorption [94]. Chemisorption occurs when electrons are exchanged or shared between the sorbate and the sorbent to form a covalent or ionic link. To put it another way, chemisorption is based on chemical interactions between the adsorbate and the adsorbent’s surface sites. Because of the strong chemical interaction between adsorbate and adsorbent, it is more difficult to reverse, and removing the adsorbed molecules requires more energy than physical adsorption [95]. Chemisorption increases with temperature at first, then reaches its maximum strength. Chemisorption is more common in heavy metal removal than other methods because it has stronger interactions and a larger adsorption capability for heavy metals.

Physisorption is a broad word that includes all weak electrostatic interactions, such as van der walls, hydrogen bonding, and dipole-dipole interactions between the sorbent and sorbate, with interactions generally ranging from 0.2 to 4 kJ/mol [95,96]. These are the weakest of the interactions and are quickly broken. When the temperature is low, physisorption occurs, and as the temperature rises, it decreases [97]. Other reported interactions include ion exchange, and precipitation. With a specified adsorbent, more than one interaction can occur during the adsorption process, but the rate and type of interactions vary due to material composition, contaminants’ structure and properties, and the solution conditions.

Adsorption techniques have some attractive characteristics, including process simplicity, cost effectiveness, resistance to toxic substances, and flexibility in the scaling-up process. The cost of the process depends upon the adsorbent material. The literature has reported [55,98,99] that adsorption is effective at removing organics, BTEX, oil, TOC, and more than 80% of heavy metals from PW, and the overall adsorption mechanism is shown in Figure 4. The activated carbon and organoclay combination has proven to be more efficient at removing total petroleum hydrocarbons (TPH) [100]. Oil content has been reduced by up to 85% in PW through the use of copolymers [101]. Zeolites have proved to be as efficient at removing BTEX compounds as the other methods [102]. Due to space constraints, efficient physical and chemical treatment technologies are preferred [13,103].

### 3.2. Factors Affecting the Adsorption Performance

The operational parameters, such as the pH of the solution, the temperature, the contact time, the mass of the adsorbent, and the surface area impact the interaction between adsorbate and adsorbent.

The pH level is most susceptible to change in adsorption investigations since H^+^ powerful absorbent. The ionization of surface functional groups and the selection of metal ions are both affected by the pH. It is considered to be one of the most critical factors affecting the binding sites, the chemical nature of the adsorbent’s surface, and the hydrogen ions. Furthermore, pH is considered to play an essential role in the adsorption system, especially in aqueous solutions, because it affects the character of each ion being removed and the adsorbents (where adsorption phenomena disappear and change to precipitation when the pH exceeds 7) [104]. Moreover, an increase in the pH enhances the pollutant removal efficiency. The removal rate of pollutants decreases at optimum pH levels. Imamoglu et al. [105] studied lead (II) and Cu (II) removal by activated carbon at different pH values, and the results revealed that many factors could be controlled by pH, such as the degree of ionization, the charge of the adsorbent material, and the specifications of the adsorbate. Krishnan et al. [106] found that the adsorbent’s pH decreased when the acidic group on the adsorbent surface increased in size. The palm tree branches were activated by acidic groups that raised the positive charge on the adsorbent surface. The adsorbent was activated by 20% and 50% H_3_PO_4_. The adsorptive capability of the adsorbent can be enhanced by changing the pH. If the pH of a solution is greater than the adsorbent pH, it provides the negative surface charge information by adsorbing cationic species. If pH < pH_pzc_ (point of zero charge PZC), it will adsorb anionic species during adsorption. Therefore, the pH of a solution has a significant effect on the adsorbent’s adsorption capacity [107].

Temperature contributes to the adsorption process. At high temperatures, the adsorption rate increases as the solution’s viscosity decreases. Adsorbate mobility also rises at a higher temperature. In physical adsorption, the removal efficiency of pollutants decreases with an increase in temperature, whereas in chemical adsorption, with an increase in temperature, adsorption increased initially and then started decreasing. Renugadevi et al. [108] studied the effects of activated carbon on methylene blue, and found that adsorption depends significantly on the temperature [109].

Contact time can be defined as the time required to achieve equilibrium. Therefore, when equilibrium is reached determines the proper contact time. Moreover, a longer contact time allows for more efficient adsorption [108].

The adsorption efficiency depends substantially on the smaller particle size and high surface area of the adsorbent. The surface area increases with the decrease in the size of the particle. An ideal adsorbent should be mechanically stable, have high active surface sites and hydrophobicity, and be eco-friendly and economically feasible [110]. Final solute concentration and the adsorption performances significantly depend on the adsorbent’s particle size. Smaller contaminant particles can adsorb more readily onto the adsorbent than larger contaminant particles [111]. Therefore, the large particles have a small surface area, causing a comparative reduction in the final uptake of the contaminants. New active sites are formed due to the increased adsorbent surface area, resulting in more solute molecules binding. Larger particles have a lower surface area, affecting the final uptake of the contaminants. New active sites are formed by increasing the adsorbent’s surface area to allow the binding of solute molecules. Matsui et al. [112] investigated the particle sizes of different zeolites and reported that the uptake of particles was 10% higher for the size of 75–100 µm than those of 150–250 µm.

Adsorbent dose plays a crucial role in increasing the adsorption rate [113]. The capacity of a solid adsorbent for a given concentration of adsorbate in a solution is usually determined by the influence of the adsorbent mass. Elsayed and Osman [104] and Mahmudi and Arsad [114] speculate that the availability of exchange sites on the surface area influences the effect of adsorbent dosage on adsorption capacity. The maximum removal of adsorbate ions from the solution was achieved before the saturation point; subsequently, no further change was observed in the amount removed. The usage of more adsorbents will therefore not affect the process of removal.

### 3.3. Conventional and Non-Conventional Adsorbents for Produced Water Treatment

**Figure 5:**
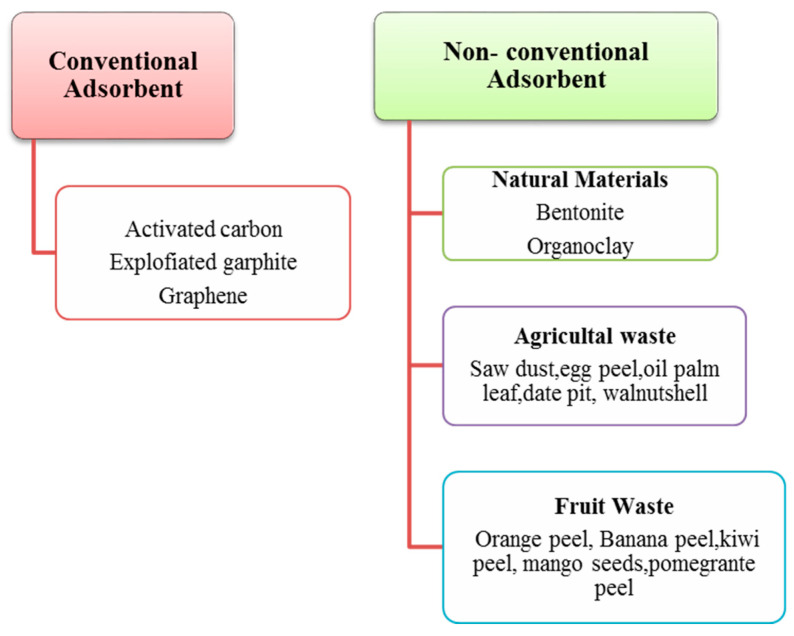
Different conventional and non-conventional adsorbents for PW treatment.

Copolymers, resins, organoclay, activated carbon, and zeolite are broadly used for water treatment. Activated carbon has an extended surface area, high surface reactivity, a microporous structure, and high adsorption ability. Functional groups are responsible for its catalytic and physicochemical characteristics. It is the most widely used adsorbent for treating wastewater; however, its use is limited because of its high cost [113]. To overcome this problem, different environmentally friendly, cost-effective, and non-conventional adsorbents have been developed for PW treatment that are made from other waste, such as fruits and plants, wood, fossil fuels, and agricultural waste [115], as shown in Figure 5.

### 3.4. Produced Water Treatment

#### 3.4.1. Oil Removal

**Figure 6:**
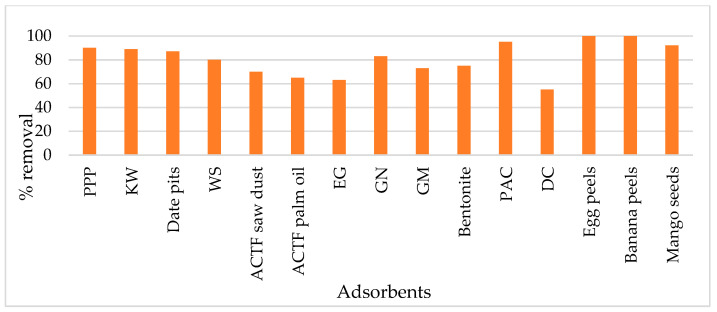
Removal of oil from produced water for the various adsorbents, including PPP (pomegranate peel powder), EG (exfoliated graphite), KW (kiwi), WS (walnut shell), GN (graphene nanoplatelets), GM (graphene magnetite), PAC (powdered activated carbon), and DC (deposit carbon).

The concentration of oil and grease (O&G) in PW ranges between 6 and 60 mg/L [116]. Johnson et al. [117] and the United States Environmental Protection Agency reported that oil and grease concentrations in PW range between 2.3 and 60 mg/L and 2.3 and 38.8 mg/L, respectively. Another study was conducted on PW in the western United States, and the concentration of O&G was found to be from 40 mg/L to as high as 2000 mg/L [23]. Different adsorbents have been used in PW for oil removal, as shown in Figure 6. Ibrahim et al. [118] used pomegranate peel powder (PPP) as an adsorbent. The results showed that the oil removal efficiency could be increased using the optimum adsorbent dosage, adsorbent concentration, and pH value. It is a low-cost adsorbent, and a 92% removal efficiency rate has been reported with a reaction time of 50 min. Another study used kiwi peel as an active and low-cost adsorbent [119]. The oil adsorption was strongly dependent on contact time, adsorbent dose, and the pH of the kiwi peels. Results revealed that an almost 90% removal efficiency rate could be achieved at a pH of 2.16 and with a contact time of 150 min for 1.5 g kiwi peels. Alsulaili and Fahim [120] used walnut shells and date pits as an adsorbent for oil removal. The results showed that oil adsorption capacities for date pits and walnut shells were 80% and 87%, respectively.

El-Syed et al. [121] investigated the use of synthesized amorphous carbon thin films that were derived from sawdust wood and utilized them for oil adsorption in a fixed-bed column system. A maximum uptake of 700 mg oil/g adsorbent was obtained at a flow rate of 0.5 mL min^−1^ and with a bed height of 5 mm. Takeuchi et al. [122] studied the use of exfoliated graphite (EG), and the result showed that 278 and 66 mg/L of oil was decreased to 1.2 mg/L and undetectable, respectively. Abou et al. [123] investigated the use of graphene magnetite and PAC for oil removal, and the results showed that the increased contact time and dosage of the adsorbent increased oil removal. The removal efficiency depends on the optimum parameters that play an essential role in adsorption. Okiel and El-Sayed [124] used powdered activated carbon, deposited carbon (DC), and bentonite, which illustrated the importance of contact time in the adsorption technique. Muhammad et al. [125] investigated the use of eggshells and achieved a 100% removal efficiency rate with a 1.8 g/L adsorbent dose.

El-Nafaty and Muhammad [126] used 267 mg/L of banana peels with 35 min of contact time and achieved a 100% oil removal efficiency rate. Another study was carried out using mango seeds that achieved a 93.3% removal efficiency rate [127]. Alther [128,129] investigated the use of organoclay, and results showed that removal efficiency is seven times higher than activated carbon and at a lower cost. It can be used as an alternative adsorbent instead of granular activated carbon. Adsorption technology with low-cost adsorbents has been shown to be good at treating oily PW to a certain extent. Oily produced water treatment is a huge problem for the oil and gas industry because it has mostly been reinjected back into the wells to increase oil production and disposal. Low-cost adsorbents, prepared from various cheap organic waste materials, can minimize waste disposal in the environment, and can also be used for pretreatment.

#### 3.4.2. Total Organic Carbon (TOC) Removal

Total organic carbon levels in PW range from 0 to 1500 (mg/L) [92,130]. Naturally occurring water has a TOC concentration between less than 0.1 mg/L and greater than 11,000 mg/L [131]. Ayers and Parker [132] found a TOC value of 300 mg/L in PW from Hibernia platforms, while TOC levels were in the range of 67–620 mg/L in PW from Louisiana rigs [133]. Gallo et al. [134] studied the adsorption properties of organic compounds, including cocoa beans, bananas, orange peel, palm shells, sawdust, and passion fruit peel, comparing them to the walnut shells that are currently the main commercially used adsorbent in PW. Before adsorption, all adsorbents undergo pretreatment, and only sawdust palm and walnut shell can be used as an adsorbent for PW. The study found that the maximum removal efficiency rate achieved using sawdust, palm shell, and walnut shell was 33, 5.6, 4.9 mg/g of the dry adsorbent, respectively. Breakthrough curves indicate that palm shell saturates much faster than walnut shell. Takeuchi et al. [122] used exfoliated graphite to remove TOC from PW and found that TOC concentrations decreased from 566 mg/L to 1.5 mg/L in the effluent.

#### 3.4.3. BTEX Removal

BTEX are volatile aromatic compounds naturally present in oil and gas products, and they can easily escape into the atmosphere during the water treatment process. Different studies have been conducted to determine the concentrations of BTEX in PW. The highest concentration was of benzene, which ranged between 0.44 and 2.80 mg/L and was reported in the PW of the Gulf of Mexico, followed by toluene, xylene, and ethylbenzene. Dorea et al. [135] investigated BTEX concentrations in Permian basin PW and found that the highest concentration was of benzene, with 1.5–778.51 mg/L, followed by ethylbenzene, xylenes, and toluene. Different adsorbents have been used for BTEX removal from PW, as shown in Table 4. In one study, organic clay was modified with a surfactant, but it was not efficient enough to remove BTEX [136]. It has a microstructure, high effectiveness, and a low cost. The outcome showed that 95.6% of the contaminant removal was achieved within 3 h, with the most-removed contaminant being ethylbenzene, followed by xylene, toluene, and benzene. Costa et al. [137] investigated the effectiveness of peat and sawdust for removing BTEX. It has been reported that peat and sawdust attained the highest efficiencies of 67% and 57% in removing xylene. A few studies used modified clay extensively for BTEX removal. Carvalho et al. [138] studied smectite clay and transformed it into an organophilic adsorbent using Na_2_CO_3_ and a hexadecyl trimethyl ammonium chloride (HDTMA) treatment. Removal rates of almost 55% and 90% were achieved. Egbuchunam et al. [139] investigated the use of surfactant-modified kaolinite (SMK) for BTEX removal from aqueous media. Kaolinite clay is an effective adsorbent due to its highly adsorptive surface area and ion-exchange properties. Sharmasarka et al. [140] investigated the use of trimethylphenylammonium (SWy-TMPA), trimethylammonium adamantane (SWy-Adam), and HDTMA montmorillonite-derivatives for BTEX removal. The results showed that the BTEX mixture’s total adsorbed amounts were higher with HDTMA than with the individual compounds.

#### 3.4.4. Metals Removal

PW contains certain metals, such as arsenic, cadmium, chromium, lead, and nickel. However, the concentration and chemical content differences are influenced by the geological age, injected water volume, and chemical composition [141]. The concentration of heavy metals in PW is often higher than in seawater. According to the Agency for Toxic Substances and Disease Registry, metals and metalloids, such as As, Cr, Cd, Ni, Cu, Zn, and Pb, are ranked as extremely toxic pollutants [142]. According to Spellman et al. [143], the removal of 85% of heavy metals could be obtained through the adsorption process. Kose et al. [144] used activated carbon for water treatment as a pretreatment technique. The results showed that metal removal from PW by the granular activated carbon microfiltration the best result using reverse osmosis. Houcine and Mejri [145] used lime in the produced water of southern Tunisia to treat heavy metals, including lead, zinc, iron, manganese, and barium. Lime proved to be an efficient and economical filtration process for heavy metal elimination, and a 95% removal efficiency rate could be achieved. According to Mahmoud et al. [118], with reed bed technology, a 78% removal efficiency rate was achieved for Al, Ba, Cr, Cu, Zn, though the rate was 40% for Fe, Li, Mn, Pb, Cd, and Ni. Another study was conducted by Fardet al. [146] for barium removal using an MXene nano adsorbent, and a 90% removal efficiency rate was attained, in which the pH was the most dominant factor. Table 4, illustrated the removal of produced water contaminates using different adsorbents. 

**Table 4:** Different types of adsorbent used for produced water treatment.

Adsorbent	Targeted Pollutant	% Removal	Limitations	References
Sawdust	COD	33%	Pre-treatment required to enhance efficiency	[134]
Walnut shell	COD	49%	Carbon is lost during reactivation	[134]
Palm shell	COD	56%	Loss of carbon during activation	[134]
Lime	Heavy metals	95%	pH dependent; produces a large amount of sludge; overdose can cause poor effluent quality	[145]
Mxene nano adsorbent	Barium	90%	Structure is not stable	[146]
Exfoliated graphite	TOC	-	Poor hydrophobicity; difficult to handle on-site because of their granular or powder forms	[122]
Peat and sawdust	BTEX	67.8% and 57.8%	Mechanical strength of peat is low, and pretreatments are required to enhance the efficiency of sawdust	[137]
Modified organoclay	BTEX	95.6%	Not suitable for pollutants that have a strongly acidic character; poor reusability and oil recovery	[138]

### 3.5. Adsorption Limitations

Activated carbon has a 70 to 85% removal efficiency rate. However, suspended contaminant particles have a low capacity and weak interaction, and there are difficulties in the regeneration of the material used as an adsorbent, while the adsorbate can decrease the removal efficiency. Moreover, the shortcomings include high installation, maintenance, and regeneration cost [147]. Another disadvantage of the adsorption process is the waste disposal requirements. After a few batch treatments, regeneration of the activated carbon is needed. Otherwise, the removal efficiency decreases significantly. Adsorbent regeneration requires various chemicals, such as organic solvents, acids, bases, and redox agents. Reactivation depends on a few factors, such as the water usage rate, contaminant concentrations, and contaminant type. The adsorbent’s surface can be modified with a hydrophilic group to enhance the surface area and porosity so as to target specific toxic materials. Several pretreatment techniques for PW adsorption treatments need to be considered in order to determine the most appropriate technique. Continuous process methods are principally eschewed at the industrial scale since they are expensive, require equipment and constant adsorbate concentrations, longer residence times, and better mass and heat transfer behavior. Overall, adsorption advantages and disadvantages are summarized in Table 5.

### 3.6. Adsorption Isotherms and Kinetics

Adsorption isotherms explain the interactions taking place between the adsorbent and adsorbate at the equilibrium stage. The interaction is based on the adsorbent, adsorbate, and solution characteristics. Contaminant removal from wastewater has become one of the major research focuses nowadays [148]. Various empirical models have been introduced to interpret the experimental data and understand the adsorption equilibrium of PW contaminants on different adsorbents, but Langmuir’s and Freundlich’s models are the most widely utilized. In the Langmuir model, adsorption is considered to be driven by a monolayer on homogeneous surfaces without interaction among the adsorbed molecules. The Freundlich model explains that multilayer adsorption occurs on a surface that has a heterogeneous distribution of active sites in the form of a monolayer.

Alsulaili and Fahim [120] studied the oil and organic pollutant adsorption properties of date pits and walnut shells. The isotherms data for the date pit fitted well to the Langmuir model, and the data for the walnut shell to the Freundlich model. Pathak et al. [113] used the Langmuir and Freundlich equations to fit the adsorption data of Cd (II), Ni, Cr, and Pb(II). The R^2^ indicated that the Langmuir equation fits better than Freundlich’s. It also suggested that adsorption on the FPW is most often monolayered. The cured oil adsorption by PPP, with an adsorption capacity of 555 mg/g, was fitted using the Langmuir isotherms [118]. Gallo et al. [134] tested three adsorption models, and found that the Langmuir model best describes the experimental data on the contaminants adsorption. The Langmuir model mostly fits the adsorption data for oil [126]. The adsorption of cured oil on powdered activated carbon, bentonite, and deposited carbon in equilibrium studies followed a Freundlich isotherm model, indicating multilayer adsorption. Increased oil adsorption was observed when the initial adsorbent dose and contact time were increased [124]. The adsorption of crude oil onto eggplant peel best fitted the Langmuir isotherm model. It can be concluded that the Langmuir model is more suitable for describing the equilibrium adsorption of heavy metals and oil in most cases. However, some studies showed that the oil adsorption dynamic data fit the Freundlich isotherm model better than Langmuir’s, as shown in Table 6.

Adsorption kinetics studies provide essential information about the rate of adsorption. The adsorption process may involve different transport stages. Adsorption kinetics explain the rate of adsorbate uptake, which physically controls the diffusion process and the residence time of adsorbate uptake at the solid-solution interface. The methods involve bulk diffusion, external mass transfer, intraparticle diffusion, and chemisorption. Three kinds of kinetic models are generally used, including the pseudo-first-order, pseudo-second-order, and Elovich models. The best-fit kinetic models for analyzing the removal of contaminants with adsorbents are summarized in Table 6. Fathy et al. [149] applied the Thomas and Yoon–Nelson models to investigate the adsorption process. The experimental data fit better to Thomas’s model. The kinetic adsorption of oil by different adsorbents was described well using a pseudo-second-order kinetic model [150]. Eggplant peel was used for cured oil adsorption, and results revealed that the data fit better to the pseudo-second-order kinetic model [151].

**Table 6:** Adsorption isotherms and kinetics models for the adsorption of contaminants from produced water.

Pollutant	Adsorbent	Isotherm Models	Kinetic Models	References
Oil and organic pollutant	Date pit	Langmuir	-	[120]
	Walnut shell	Freundlich	-	
Heavy metals	Fruit peel waste	Langmuir	Pseudo-second order	[113]
Oil	Pomegranate peel	Langmuir	Pseudo-second order	[118]
Oil	Amorphous carbon thin film (palm oil)	-	Thomas model	[149]
Oil	Banana peels	Langmuir	Pseudo-second order	[126]
Oil	Bentonite, PAC, and DC	Freundlich	-	[124]
Oil	Eggshells	-	Pseudo-second order	[125]
Oil	Eggplant peel	Langmuir	Pseudo-second order	[151]

## 4. Recent Progress in the Development of Porous Adsorbent

Research has been conducted to overcome the shortcomings of conventional adsorbents by synthesizing porous materials that have large pore volumes, good pore size distributions, large surface areas, and regular structures onto zeolites, activated carbon, metal oxide, and mesoporous clay [151,152]. Zeolites have a narrow pore size, whereas metal oxides do not have a large surface area. These materials are not feasible at a large scale because of the stability and leaching of toxic metals into water bodies [153]. Activated carbons and zeolites are hard to synthesize due to the pores’ tuning properties. Moreover, they have been used for liquid-phase water treatment due to the large surface area.

### 4.1. Metal–Organic Frameworks (MOFs)

In recent years, MOFs have become one of the most attractive porous crystalline materials, and they have been intensively investigated as an excellent alternative to other methods, given that they can overcome the limitations of conventional porous materials and give promising results in adsorption-related applications [147]. MOFs are a combination of organic and inorganic materials. The history of MOFs dates back to the early 1990s. Kanoo et al. [139,154] developed a coordination polymer, in which a metal node made of copper (I) and an organic linker consisting of nitrogen were used. The synthesized 3D polymer was introduced for the first time to a coordination polymer, which laid the foundation for the advanced development of MOFs. Fujita and Kwon [155] formed a two-dimensional square network composed of cadmium and 4,4′ bipyridine. Organic components in this material surrounded the inner cavities. In 1995, Yaghi, Li [156] synthesized a porous material consisting of organic molecules and metal ions. In this study, a newly synthesized material was developed that could tolerate temperatures of up to 350 °C even after removing the guest molecule. Experimental results proved that the channels were permanent. Yaghi successfully proposed the MOF concept, and this is a landmark in the history of MOF development. Li et al. [157] famously synthesized a highly porous and thermally stable MOF-5 with a large surface area, and successfully stored methane in it. This unique material has now gained the attention of scientists globally for the synthesis of new composites, and for gas storage applications.

### 4.2. The Significance of Metal–Organic Frameworks

Metal–organic frameworks are expected to be the best high-capacity adsorbent due to their high porosity, large surface area, and different configuration and structure. They have several advantageous features, such as great adsorption sites, regular and tunable pore structures, functional pore space, and large surface areas. This new class of materials are used in adsorption and have many promising applications, such as catalysis [158,159], drug delivery [160], small molecules sensing gas adsorption [161], and separation [162]. Above all, regeneration and stability are some of the main criteria for the selection of adsorbents. An important advantage of MOFs over traditional adsorbents (such as zeolites and activated carbons) is that the pore environment of the MOFs can be modified, which gives users control over the structural properties that are required for any specific application [163]. Porosity and nanopore diameter are considered to be the key parameters for potential MOF applications because they facilitate the penetration of adsorbed substances to the inner space. MOFs have a great diversity of use due to their inorganic clusters, pore sizes, chemical functionalities, and pore structures.

**Figure 7:**
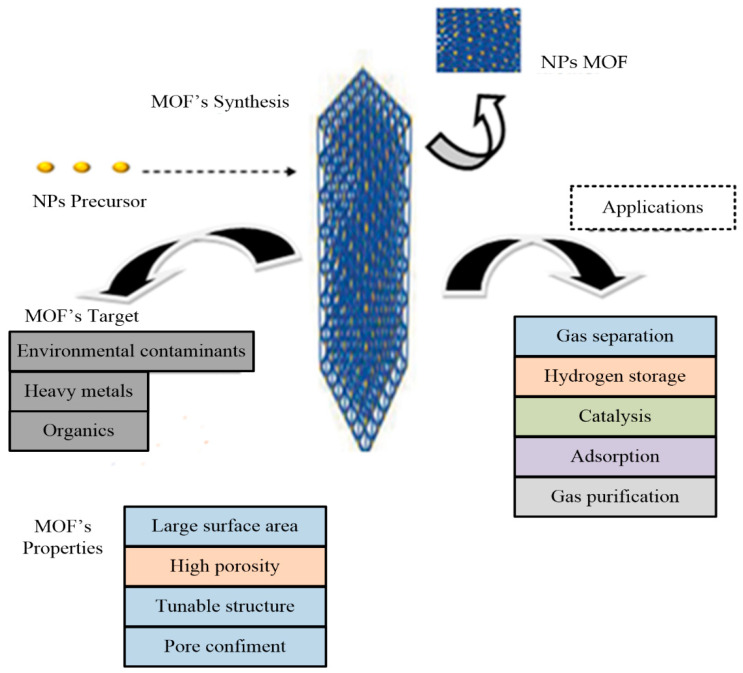
Schematic diagram of MOF properties and applications.

In the 21st century, nanotechnology has introduced specific, ultrahigh surface area, high-intensity, small-sized nanomaterials to the water industry that have dimensions which range from the sub-nanometers to several hundred nanometers [164,165]. However, the problem with nanoparticles’ high surface-to-volume ratio is the intrinsic instability that limits nanoparticles’ broad applicability. Different materials, such as zeolites and mesoporous, have been used, but recently MOFs were found to be a better stabilizer. Introducing specific nanoparticles into the permanent porous structure of MOFs results in a more stable material [166]. MOFs act as the precursor or template on which to include nanoparticles, which can fit in their cavities to create a composite made of MOFs and nanoparticles. These hybrid materials have a much lower density and a superior thermal stability [167]. Many efforts have been made to ensure the better fabrication and greater applicability of composites [168,169,170]. The composites of MOFs and nanoparticles are used as hydrogen storage materials [171,172], catalysts [173], acidic gases adsorbents, ammonia, and as separator in the batteries that are made up of lithium–sulfur. Furthermore, MOFs have been used widely for wastewater treatment. To date, it is rarely used for PW treatment, however. Yet this advanced method is suggested as the future of PW treatment, as shown by the schematic diagram in Figure 7.

### 4.3. Metal–Organic Frameworks as Adsorbents

MOFs have been widely used for water regeneration and wastewater treatment, and it is highly recommended that they are used for PW treatment in the future. As an adsorbent, MOFs are cost effective, with no sludge generation and no additional chemicals required, other than conventional adsorbents. MOFs can regenerate without changing their properties. The current literature on wastewater shows that MOFs provide much better performance than other regular porous materials. Based on these studies, the application of water-based MOFs as an adsorbent in the field of gas or liquid phase adsorptions has been suggested. This will allow a variety of applications of MOFs. Hydrophilic mesoporous compounds were found to be the most favorable of the water-stable MOFs. The water adsorption of porous materials is becoming tremendously important in thermal batteries, water delivery in remote areas, and dehumidification applications. MOFs’ structural features, such as excellent BET surface area, topologically large pore volume, size, and the presence of hydrophilic functional groups as adsorption sites, make it very effective for pollutant uptake, especially at low pressures.

Furukawa et al. [174] has identified three criteria that need to be met for MOFs applications to be considered useful:

- Water condensation in the solid pores shows steep uptake behavior.
- Facile desorption and adsorption for energy efficiency and a high uptake capacity for water.
- High water stability and cycling performance.

Furthermore, MOF-based adsorbents have promising applications in adsorbing specific contaminants from water environments, as shown in Table 7. Water-stable MOFs could effectively target compounds in water systems, including dyes, drugs, pharmaceuticals, organic chemicals, and metal ions. MOFs have a superior surface area and more active sites compared to the conventional adsorbent. Nonetheless, MOFs, as porous coordination materials, have excellent chemical stabilities under different harsh conditions that make them well suited for targeted applications. Water-stable MOFs could be one of the most powerful adsorbent materials due to their good chemical stability and their contributions to an energy-efficient and cost-effective separation process.

#### 4.3.1. Adsorption of Organics

Water-stable MOFs have the potential to adsorb organics from wastewater. Yang et al. [175] developed fluorous metal–organic frameworks (FMOFs) with superhydrophobicity and which showed significant air and water stability. These FMOFs adsorbed the C_6_–C_8_ hydrocarbons of oil components. Such MOFs can thus be utilized for hydrocarbon storage and cleaning oil spills. Chun et al. [176] developed metal–organic framework for microporous organic network (MOF@MON) hybrid materials that showed exceptional performance in the adsorption of toluene from water. Xie et al. [177] performed a wide-ranging study to screen a series of aluminum-based MOFs, CAU^−1^, and MIL-68(Al) for the adsorption of nitrobenzene from water. The MOFs’ excellent stability and reusability revealed that they are promising adsorbents for the effective adsorption of organics from wastewater.

Jin et al. [178,179] studied different zirconium-based (ZIF-based) MOFs for the adsorption of 5-hydroxymethylfurfural (HMF). The result showed that HMF uptake at equilibrium increased following in integration of ZIF-93, ZIF-90, and ZIF-8. The results proved that ZIF-8 could be an efficient and reusable adsorbent for HMF recovery from aqueous solutions. Seo et al. [179] used UiO-66 to remove a herbicide and found that UiO-66 had a very high-level adsorption rate, especially with low concentrations of methylchlorophenoxypropionic acid (MCPP) compared to the activated carbon. Electrostatic and p–p interactions were essential in the overall adsorption process.

#### 4.3.2. Adsorption of Heavy Metals

Inorganic ion removal is essential for two primary reasons:

- Precious metal collection and recovery of these ions can contribute to the progress of their applications in industries.
- As hazardous pollutants, they can have serious negative health effects on human beings and ultimately could be a major global threat to the environment.

Highly water-stable MOF structures have recently been developed and considered to be robust adsorbents for metal adsorption from wastewater [180,181]. Chowdhury et al. [182] synthesized highly stable and hydrolysis-resistant aluminum-based MOF-GO nanocomposites. The adsorption of As (III), chosen due to low density and high surface area, onto MIL-53(Al) was studied [183]. The results showed that textural properties could be modified by altering the GO to MIL-53(Al) mass ratio. Importantly, MIL-53(Al)-GO has a higher As(III) adsorption capacity compared to individual moieties. A new, thermally stable, 3D MOF, made from cobalt ion, was synthesized by Abbasi et al. [184], and was applied to the adsorption of Pb^2+^, Hg^2+^, Al^2+^, Fe^3+^, and Cd^2+^ metal ions from wastewater. This study investigated the effect of exposure time and pH on metal adsorption. Results showed that maximum removal was achieved for Fe^3+^ and other metal ions after 80 and 100 min. One of the critical parameters that affects the uptake of metal ions from wastewater is pH, and the highest removal rate of the metals was obtained by when the pH increased from 2 to 6. Another study was conducted to synthesize chemically stable and reusable MOF-88 nanoparticles for arsenic adsorption from wastewater [185]. It had a 24.83 mg/g adsorption capacity. Ke et al. [180] studied a Cu-based MOF for the adsorption of Hg^2+^ that was functionalized through coordination bonding between the thiol groups of dithioglycol and the CUS of Cu-BTC. The functionalized thiol exhibited a high adsorption capacity (714 mg g^−1^) for Hg^2+^, whereas the non-functionalized Cu-BTC had no Hg^2+^ adsorption capacity under the same experimental conditions. High adsorption on the porous MOF’s inner surface was found to be due to the high density of the thiol groups [186,187]. Moreover, a study suggested that MOFs could be used with the ion-exchange method for metal adsorption. It has been observed in a few cases that a simple ion-exchange method leads to the formation of a few iso-structural MOFs [188,189]. Zhu et al. [181] synthesized an iron-based MIL-100(Fe) MOF and found that it could remove arsenic (As). The adsorption of arsenic on MIL-100(Fe) pH was studied, and the adsorption was reported at a wide pH range (2–12). The results showed at optimum pH of 4, where a 98.2% removal efficiency rate was achieved, while above pH 12, the efficiency decreased drastically to 35% because MOF is not stable in basic conditions. No pH adjustment is needed for neutral water treatment as its pH ranges between 6–8.5. Arsenic adsorption onto MIL-100(Fe) was six and 36 times superior to iron oxide nanoparticles and commercial iron oxide powders, respectively. Table 7 illustrates the efficiencies of MOF for wastewater treatment.

**Table 7:** Metal–organic framework for wastewater treatment.

MOFs	Pollutants	Removal Efficiency	References
MIL-53(Al)-GO	As (III)	94.8%	[182]
3D Cobalt MOF	Pb^2+^ Hg^2+,^ Al^2+,^ Fe3 Cd^2+^	-	[184]
MOF-808	As	80.07%	[185]
MIL-100(Fe)	As	98.2%	[181]
Cu-BTC	Hg^2+^	90.74%	[180]
MIL-96	Arsenic	80%	[190]
FMOF-1	FMOF-1	87.7%	[175]
ZIF-8	Hydroxymethylfurfura	96.8%	[191]
UiO-66-NH2@MON	Toluene	87.3%	[192]
UiO-66	Methylchlorophenoxypropionic acid	98.7%	[179]

## 5. MOF Recycling

MOF recycling and reuse is necessary for industrial application. MOF generation requires metal salts, linkers, and solvents. The processing itself is expensive, but organic linkers are the key expense among such consumables. MOF waste recycling depends on its solvent content, thermal and chemical stability, chemical bonding, and structure. The high cost of resources and its toxic and harmful effects on the overall environment could be reduced by recycling the used solvent. MOF recycling is crucial for a sustainable environment, and particularly for energy and resource conservation. Recycling MOFs contains three steps: waste MOF collection, processing, and the production of new products [193,194,195]. Recycling MOFs can reduce waste generation, prevent pollution, and provide economic security. Cost-effective adsorption and desorption are the fundamental characteristics of good adsorbent material. The regeneration of the adsorbent helps to improve the economic feasibility of the process and enhances the possibility of using the adsorbents on a commercial scale. During massive adsorption–desorption regeneration, cyclic stability is an important parameter to determine the lifespan of the MOF and decide whether it should be replaced in the adsorption plant. After the multi-stage cycle, the adsorbent’s sorption capacity should not be reduced, nor should it change its structure and properties. Thermogravimetric methods are frequently used to determine adsorbent sorption capacity, renewability, and stability. Only the guest molecules could be removed from a porous framework during regeneration while keeping the structures intact through activation. During activation, accessible pores and open metal sites are produced to improve how the guest molecules interact with the host in MOFs. Ideally, the MOF’s structural and chemical properties must be retained during the regeneration and reuse process.

## 6. Future Research Perspectives

The upward flow of a large volume of PW during oil and gas production is becoming a major problem for the oil and gas industries. PW is highly polluted with immiscible oil, organic compounds, heavy metals, salts, suspended solids, and radioactive waste, which need to be removed from PW on site before letting it enter the environment. Based on the literature reviewed, the conventional treatment methods display poor performance and have other significant limitations, such as prolonged treatment time, vast land requirements, and high capital costs. Most importantly, the traditional treatment approaches produce secondary pollutants which need further treatment, making the conventional process less attractive in the long run. The most attractive and feasible treatment approach could be adsorption, but only if the challenges faced by this technology are overcome. Its efficacy has been proven in previous studies. However, the adsorbents used in adsorption studies require a critical assessment. Adsorption provides a cheaper alternative for the remediation of PW.

Nevertheless, the factors affecting the performance of adsorbents, such as surface area, adsorbent dosage, treatment temperature, contact time, and pH, to name a few, need to be controlled and optimized to maximize PW treatment efficiency. MOFs are understood to offer better alternatives for PW water treatment than traditional adsorbents. For example, MOFs can tolerate high temperatures and provide a large surface area, and have greater porosity and better nanopore diameters. It is suggested that highly porous and new hybrid MOFs could be developed and used for PW treatment in large-scale applications. Since the PW is contaminated with various organic and inorganic compounds, future research should produce MOFs that simultaneously remove multiple contaminants. Future studies should also consider the regeneration of MOFs at low temperatures, reducing the overall cost of PW treatment. New routes for synthesizing MOFs could consider eliminating the need for expensive chemicals and solvents and move towards a green and sustainable process.

## 7. Conclusions

Adsorption performed an essential role in removing contaminants. As a result, it has been the focus of significant amounts of attention in both scientific research and commercial applications. This review has summarized the currently investigated conventional and non-conventional adsorbents for PW contaminates. Since PW generation is a fundamental problem, more investigation is required to develop techniques to reduce PW volumes. Studies on adsorption for PW treatment are limited, and there is a lack of a comprehensive understanding regarding adsorption applicability and the strategies to improve the treatment efficacy. The most attractive and feasible treatment approach could be adsorption, if the challenges faced by this technology are overcome. The safest technology for treating PW depends on its chemistry, cost effectiveness, space availability, reuse and discharge plans, durable operation, and byproducts. Furthermore, the development of highly porous, efficient, eco-friendly, cost-effective, mechanically stable, and sustainable adsorbents is the main concern for adsorption to overcome its limitations, which include low surface areas and restrictions on reuse and regeneration, and which limit its application at a large scale. Ideal adsorbents will minimize stress on freshwater resources, and have a range of positive features, including availability, non-toxicity, cost, metal-binding capacity, and regeneration as part of their application in PW treatment. This is particularly important for water-stressed countries, where population and economic growth continue to increase stress on the region’s limited water resources.

## Figures and Tables

**Table 1:** Composition of produced water [3].

Parameter	Units	Ranges
pH	-	4.3–10
Total Dissolved Solids	mg/L	100–400,000
Total Suspended Solids	mg/L	1.2–1000
Chemical Oxygen Demand	mg/L	1220–2600
Total Organic Carbon	-	1500
Salinity	mg/L	5000–300,000
Conductivity	μS/cm	4200–58,600
Surface Tension	dyn/cm	43–78
Density	kg/m^3^	1014–1140

**Table 2:** Concentration of TENORMS in produced water from oilfields around the world.

TENORMS	Concentration Bq. L-1	Countries	References
226 Ra	5.1–14.8	Algeria	[26,27,28]
	0.5–16	Norway	[22]
	13.8–111.2	Syria	[29]
	1.07–34.15, 5–40	Egypt	[30,31]
	(<0.002–58)	USA	[32,33]
210 Pb	<5	Poland	[34,35]
	2.6–16.7	USA	[32,33]
228 Ra	<0.05–12.0	Brazil	[28,36]
	<0.02–13.26	Egypt	[32,33,37,38,39]
	<2	Poland	[34]
	<1–4	Turkey	[40]
	6.40–35.50	Ghana	[37,41]
	8.1	Nigeria	[31,38]
	<1.1 × 10^−3^–9.6	Argentina	[42]
	35–763, 0.02–59	USA	[32,33]
40 K	39.8	Nigeria	[31,38]
	1.65–11.99	Ghana	[37,41]
	1522–1535	Oman	[43]
	221–899	Romania	[43]
	4.4–43.7 632.5–1448.7	Egypt	[28,30]
	14.6	Iraq	[44,45]
	3.6–15.37	Azerbaijan	[29]
	7.3	Iran	[46]
238 U	<4.5 × 10^−3^	Congo	[41,42,46,47,48]
	7.3 × 10^−3^–1.5 × 10^−2^	Italy	[49]
	9.47–25.2	Egypt	[28,30]
	4.12	Iraq	[44,45]
	0.043–1.1	Ghana	[37,41]

**Table 3:** Produced water effluents discharge limit for different countries [84,85].

Country	Effluent Limits		Reporting Routine
	Monthly	Daily	
Canada	40 ppm monthly avg.	80 ppm 2-day avg	Annual
USA	29 mg/L monthly avg.	42 mg/L daily max	Monthly
UK	40 ppm monthly avg.	-	Annual
Western Australia	30 ppm monthly avg.	50 mg/L daily max	-
Mediterranean Sea	40 ppm monthly avg.	-	-

**Table 5:** Advantages and disadvantages of adsorption.

Advantages	Disadvantages
It is feasible for all the contaminants present in PW	It cannot remove TDS and salt concentrations
It can considerably reduce TOC, BTEX, and oil concentrations	For media regeneration, expensive chemicals are required
It is used as a polishing step in PW to achieve the best results	It cannot be used as a major treatment process due to the rapid consumption of adsorbent material
It uses compact, packed bed modules, and is cheaper, efficient, and requires minimal energy	A disposal system is required for waste generated by used adsorbent media, or some form of regeneration
It can remove 80% of heavy metals	It has a high retention time
Nearly 100% of water recovery can be achieved	Less efficient at a higher feed concentration

## Data Availability

Not applicable.

## Abbreviations

Ac	Actinium
BOD	Biological oxygen demand
Cd	Cadmium
COD	Chemical oxygen demand
Cr	Chromium
Cu	Copper
DC	Deposited carbon
EG	Exfoliated graphite
FMOFs	Fluorous metal–organic frameworks
GM	Graphene magnetite
GN	Graphene nanoplatelets
HMF	Hydroxymethylfurfural
KW	Kiwi
MCPP	Methylchlorophenoxypropionic acid
MOFs	Metal–organic frameworks
NPD	Naphthalene, phenanthrene, dibenzothiophene
OIW	Non-polar oil in water
PAC	Powdered activated carbon
PPP	Pomegranate peel powder
PZC	Point of zero charge
Ra	Radium
TDS	Total dissolved solids
TENORM	Technologically enhanced naturally occurring radioactive materials
TOC	Total organic carbon
TSS	Total suspended solids
TOC	Total organic carbon
WS	Walnut shell
Zn	Zinc
